# Effects of acute bovine colostrum supplementation on immune responses to prolonged cycling: a randomised crossover trial

**DOI:** 10.1007/s00394-026-04016-5

**Published:** 2026-06-06

**Authors:** Arwel W. Jones, Rhys Thatcher, Glen Davison

**Affiliations:** 1 School of Translational Medicine, Monash University, Melbourne, Australia; 2 Department of Life Sciences, Aberystwyth University, Aberystwyth, UK; 3 School of Natural Sciences, University of Kent, Canterbury, UK

**Keywords:** Immune function, Leukocyte, Saliva, Neutrophil, Antimicrobial peptides, Exercise

## Abstract

**Purpose:**

The purpose of this double-blind placebo-controlled crossover trial was to assess the effects of acute bovine colostrum supplementation on exercise-induced changes in neutrophil function and mucosal immunity following prolonged exercise.

**Methods:**

Sixteen healthy, recreationally active males participated in two trials in a randomised counterbalanced order. Participants either consumed doses of bovine colostrum or an isoenergetic placebo 1 h (30 g) and immediately prior (5 g) to 2.5 h of cycling at 15% Δ with a further dose midway through the exercise (5 g). Venous blood and unstimulated saliva samples were obtained at baseline, 1 h post-consumption (pre-exercise), immediately post-exercise and 1 h post-exercise. A mechanistic sub-study was performed to determine whether plasma obtained from participants following bovine colostrum consumption directly enhances neutrophil function.

**Results:**

Compared to placebo, a greater fMLP-stimulated blood neutrophil oxidative burst was observed (~ 15% difference across timepoints) with bovine colostrum (trial, *p* < 0.05). In the sub-study, fMLP-stimulated neutrophil oxidative burst was enhanced when whole blood was pre-incubated with plasma obtained 1 h following colostrum consumption. There was greater salivary lysozyme concentration and bacterial-stimulated blood neutrophil elastase release at post-exercise and 1 h post-exercise respectively in the bovine colostrum trial (trial × time interaction, *p* < 0.05). There was no effect of bovine colostrum on leukocyte trafficking, PMA-stimulated neutrophil oxidative burst, salivary secretory immunoglobulin A and salivary lactoferrin.

**Conclusion:**

Acute bovine colostrum supplementation can modify innate immune responses to prolonged exercise, which may be due to components or metabolites of bovine colostrum that become bioavailable following consumption.

**Supplementary Information:**

The online version contains supplementary material available at 10.1007/s00394-026-04016-5.

## Introduction

Strenuous, prolonged exercise results in significant transient perturbations of immune function which includes, but is not limited to, decreases in both cell-mediated (e.g. neutrophil oxidative burst activity, skin delayed-type hypersensitivity response) and mucosal parameters (e.g. salivary immunoglobulin A, IgA) [1]. It has long been proposed that such immune perturbations increase the susceptibility to upper respiratory illness, particularly under repeated cycles of strenuous exercise or when this exertion is combined with other stressors (e.g. lack of sleep, inadequate nutrition) [2, 7]. Various nutritional interventions have been investigated as strategies to minimise exercise-induce immune dysfunction and risk of upper respiratory illness but many lack evidence to support their use [3].

Bovine colostrum is the initial milk produced by a cow following parturition and made commercially available as a supplement by the dairy industry to promote general health and immune support [4]. Daily bovine colostrum supplementation (10–20 g) for 8–12 weeks results in a clinically relevant reduction in the number of days and episodes of symptoms of upper respiratory illness [5]. Previous work in our laboratory has also shown that 4 weeks of bovine colostrum (20 g day^−1^) supplementation can limit the immunodepressive effects of an acute physical stressor (2 h of running) on in vivo immune responsiveness to a novel antigen [6]. Given the observed effects of bovine colostrum on clinically relevant endpoints, further studies with ex vivo measures of immunity are now important to advance our understanding and provide additional mechanistic information [7]. Daily bovine colostrum supplementation for 4 weeks does counter the effects of prolonged exercise (2–2.5 h of cycling) on ex vivo measures of innate immune cell function (neutrophil oxidative burst, neutrophil stimulated-degranulation) [8, 9]. The mechanism(s) underlying the effects of bovine colostrum remain unclear. Importantly, the available evidence does not show any consistent measurable effects of daily supplementation on resting immune measures during periods of exercise training [10]. Bovine colostrum consumption is suggested to result in bioactive components becoming biologically available to influence immune responses, and such mechanisms may be evident in shorter periods, such as after acute consumption before or during exercise [11].

Animal and in vitro cell culture studies have demonstrated direct effects of bovine colostrum on phagocytosis and oxidative burst of neutrophils [12–15], whereby short-term exposure of cells to bovine colostrum amplified their response to subsequent activation. Sugisawa et al. [15] proposed that such “priming” is mediated by low-molecular weight substances (< 10 kDa) present in bovine colostrum. In an investigation of healthy humans at rest, Jensen et al. [16], demonstrated that phagocytic activity of neutrophils was enhanced 1 h following a single oral dose of a low-molecular weight fraction of bovine colostrum. To our knowledge, despite such evidence of bovine colostrum and its components, no studies have investigated the effect of acute bovine colostrum supplementation on exercise-induced changes in neutrophil function.

Mucosal immunity, with assessment of saliva secretory IgA (SIgA) and salivary antimicrobial peptides, have been areas of investigation with daily bovine colostrum supplementation periods of 8 days to 12 weeks [8, 9, 17]. Large variability between participants (parallel group designs) and the limited use of acute models of exercise stress (providing greater need for intervention) have been recognised as important limitations for investigating the effect of bovine colostrum on mucosal responses [9]. The lack of studies on shorter periods (< 4 weeks) and higher doses of bovine colostrum (> 20 g) on salivary SIgA and antimicrobial peptides provide further unanswered questions. Given the role of neutrophils as a source of antimicrobial peptides [18], any acute, direct effects on neutrophil capacity may also lead to improvements in innate mucosal responses [19].

It is currently unknown whether priming and ‘immune-enhancing’ effects of bovine colostrum will present with acute oral supplementation in a model of exercise-induced immune dysfunction. Hence, the primary aim of this study was to determine the influence of acute oral bovine colostrum supplementation on blood neutrophil oxidative burst. We performed a mechanistic sub-study to determine if plasma obtained following bovine colostrum consumption was able to enhance neutrophil function in placebo samples. This would extend previous in vitro culture studies of direct exposure of cells to bovine colostrum and determine if biologically active components or metabolites of bovine colostrum that appear in the circulation following consumption (in vivo) are a potential explanation for any observed effects on neutrophil function. Our secondary aim was to determine the effect of acute oral bovine colostrum supplementation on blood neutrophil degranulation and mucosal immunity following prolonged exercise.

## Methods

### Design

This was a double-blind placebo-controlled randomised crossover trial. Ethical approval for the study was granted by the Department of Sport and Exercise Science Research Ethics Committee at Aberystwyth University, and all procedures were conducted in accordance with the Declaration of Helsinki principles. All participants provided both verbal and written consent following information on experimental procedures. We included healthy, recreationally active (self-reported) males (with experience of cycling) aged between 18 and 45 years. Exclusion criteria were as follows: exhibiting risk factors for cardiovascular, metabolic or any other disorders exacerbated by involvement in prolonged exercise; incidence of respiratory infection in the 4 weeks prior to the study; allergies/intolerant to any dairy food products; current use of any medications or dietary supplements; current smoker, and donated blood in the 4 weeks prior to the study. Participants were required to complete a physical activity readiness questionnaire on any study visit involving exercise.

### Randomisation and blinding

The allocation and counter-balanced order of experimental trials (involving acute supplementation with bovine colostrum or a placebo) was completed using an online randomisation tool (randomization.com). The randomisation schedule was generated by a researcher not involved in data collection. To maintain double-blinding, bovine colostrum and placebo powders were mixed into solutions (at a ratio of 1 g to 10 ml of water and combined with vanilla flavouring (MP flavouring, My Protein, Northwich, UK) by a technician independent from the research team. Solutions were indistinguishable in both taste and appearance and were supplied to the investigators on the morning of each day of testing. Blinding was maintained until after final statistical analyses.

### Supplementation

The bovine colostrum and placebo supplement for this study were identical to those used in our previous studies [8]. The bovine colostrum supplement (powder) was provided by Neovite UK, London. The placebo supplement was an isoenergetic and isomacronutrient mixture of milk protein concentrate and skimmed milk powder (My Protein, Northwich, UK).

### Cardiopulmonary exercise test and familiarisation trial

As previously described [9], gas exchange threshold (GET) and $\dot{\mathrm{V}}{\mathrm{O}}_{2}$ max were determined via a continuous incremental test (30 W min^−1^ ramp rate following 3 min of unloaded baseline pedalling) to volitional exhaustion on an electrically braked cycle ergometer (Lode Excalibur, Groningen, The Netherlands). For each participant $\dot{\mathrm{V}}{\mathrm{O}}_{2}$ max and GET were used to estimate an exercise intensity (power output) that would elicit 15% Δ (15% of the difference between power output at GET and $\dot{\mathrm{V}}{\mathrm{O}}_{2}$ max), equivalent to ~ 55–60% of the participant’s $\dot{\mathrm{V}}{\mathrm{O}}_{2}$ max. A familiarisation trial took place seven days following the incremental test to accustom participants to the testing procedures and physical stress expected in the main experimental trials. Participants performed a 2.5 h exercise bout on the electronically braked cycle ergometer at an intensity of 15% Δ. Expired gas was analysed during the 10th, 30th, 60th, 90th and 120th min of exercise to verify that the selected work rate did elicit the target intensity. Heart rate (HR) was monitored every 15 min using a telemetric device (Polar S610, Polar Electro Oy, Kempele, Finland). RPE was monitored every 15 min using the Borg scale [20].

### Main experimental trials

At least seven days following the familiarisation trial participants performed two trials separated by seven days in a counterbalanced design. All participants were asked to refrain from heavy exercise and consumption of alcohol during the 48 h prior to the main experimental trials. All participants were asked to keep a written weighed food diary for the 24 h prior the first main experimental trial and follow that exact diet during the 24 h prior the second experimental trial. On the day of each main experimental trial, participants completed a printed food frequency questionnaire relating to the previous seven days. The dietary records were analysed using computer dietary analysis software (CompEat, Nutrition Systems, London, UK).

On the morning of each trial, participants reported to the laboratory after an overnight fast of at least 10 h. Participants were asked to consume 500 mL of water 2 h before arrival to support euhydration. This was confirmed verbally on arrival. Participants were then asked to remain seated for 10 min prior to collection of a resting Baseline blood sample from an antecubital vein and an unstimulated saliva sample. In randomised counterbalanced order, participants then consumed a solution containing 30 g of bovine colostrum for one trial while the other trial involved the consumption of 30 g of placebo. Participants undertook restful activities (i.e. reading) in the laboratory before collection of a Pre-exercise venous blood sample and unstimulated saliva sample at 1 h post consumption of bovine colostrum or placebo. Immediately prior to the exercise participants consumed a further 5 g of bovine colostrum or placebo mixed in 50 mL of water and vanilla flavouring.

All participants also received a further 5 g of bovine colostrum or placebo (in 50 mL water) at 1 h 15 min into the exercise of the respective trial. Except for this mid-way point, participants were permitted diluted cordial (four volumes of water to 1 volume of sugar-free cordial at 2 mL kg^−1^ of BM) during the first trial with the pattern of intake and total volume replicated in the second trial. As a replication of the familiarisation trial, expired gas was analysed during the 30th, 60th, 90th and 120th min of exercise, with HR and RPE monitored every 15 min of the 2.5 h of cycling. The mean HR and RPE were used to determine whether a similar physical stress was imposed in each experimental trial. A venous blood sample (one K_3_EDTA and one L-heparin vacutainer) and an unstimulated saliva sample were collected immediately Post-exercise. Participants remained fasted until a further blood and saliva sample were obtained at 1 h Post-exercise.

### Blood sample processing and analyses

Haemoglobin, total and differential leukocyte counts were measured in each K_3_EDTA vacutainer using an automated haematology analyser (Pentra 60 C+ Haematology analyser, HORIBA Medical, Montpellier, France). Haematocrit was determined from an aliquot of whole blood (heparin anti-coagulated) by a standard microcentrifugation method (using a Hawksley microcentrifuge). This was used along with the previously attained haemoglobin concentration, to estimate changes in blood and plasma volume from pre- to post-exercise as previously described [21]. The remaining blood in K_3_EDTA and heparin vacutainers were centrifuged at 1500 g for 10 min at 4 °C with subsequent plasma being stored at -80 °C.

As previously described [8], whole blood from K_3_EDTA treated tubes was used to determine in vitro stimulated neutrophil oxidative burst response to fMLP and PMA using a commercially available chemiluminescence kit (ABEL, Knight Scientific Ltd, Plymouth, UK). The chemiluminescence per well was measured by a microplate luminometer (FLUOstar OPTIMA, BMG Labtech, Aylesbury, UK). To account for oxidative burst responses on a per cell basis, it was assumed that the chemiluminescence responses were attributable largely to the neutrophils within the samples [22]. Thus, PMA- and fMLP-stimulated responses were divided by the number of neutrophils present in each well to give chemiluminescence in RLU (i.e. oxidative burst response) per neutrophil. To facilitate inter-subject comparisons, Pre-exercise, Post-exercise and 1 h Post-exercise stimulated oxidative burst responses were expressed as a percentage of the Baseline value, in accordance with previous trials [9].

In accordance with previous methods [8, 9], heparinised blood was used to determine neutrophil degranulation responses to bacterial stimulant (840-15, Sigma, Poole, UK). Bacterial-stimulated degranulation was based on measuring the amount of stimulated elastase release per neutrophil using an ELISA kit (Merck Calibiochem, Darmstadt, Germany) at Pre-Exercise, Post-exercise and 1 h Post-exercise.

### Mechanistic sub-study: incubation of plasma following bovine colostrum or placebo consumption with whole blood

To test the hypothesis of direct enhancement of neutrophil oxidative burst by components or metabolites that become bioavailable in the circulation following consumption of bovine colostrum, we examined the effect of incubating plasma (with or without bovine colostrum consumption) on fMLP-stimulated oxidative burst in whole blood collected at baseline and post-exercise timepoints of the experimental trials. A subset of participants (*n* = 7) were asked to attend the laboratory for two visits separated by seven days prior to the start of the main experimental trials. On each visit participants consumed either 30 g of bovine colostrum or placebo (solutions prepared as in the main experimental trials). K_3_EDTA anti-coagulated blood obtained 1 h following consumption of these solutions was immediately centrifuged at 1500 *g* for 10 min at 4 °C with subsequent plasma stored at -80 °C so that any components or metabolites that were bioavailable in this plasma (i.e. after digestion and absorption of the ingested product) could be persevered for later incubations with fresh blood in the experimental trials. At each experimental trial performed by this subset of participants, an aliquot of their stored plasma obtained 1 h following bovine colostrum and placebo consumption was thawed. Once thawed plasma was incubated with whole blood (1:1 ratio, 100 µl each) obtained at Baseline and Post-Exercise. Following an incubation period of 30 min at 37 °C, blood was immediately analysed for fMLP-stimulated oxidative burst. Upon unblinding of trials, the effect of the plasma on neutrophil activation was determined using the Baseline and Post-exercise timepoints of the placebo (control) main experimental trial only.

### Saliva sample processing and analyses

Following collection (Baseline, Pre-exercise, Post-Exercise, 1 h Post-Exercise), all saliva samples were centrifuged for 5 min at 16,000 *g* to pellet debris leaving the remaining clear supernatant to be aliquoted and stored at – 80 °C for later analysis. The unstimulated collection period was adjusted if necessary to ensure sufficient volume for all analyses (at least 300 µl, maximum 3 ml), with collection periods being a minimum of 2 min and maximum of 6 min. All saliva samples were thawed at room temperature only once prior to analysis. Upon thawing of saliva, samples were again centrifuged for 5 min at 16,000 *g* to precipitate mucins and other debris and allow for the resulting clear supernatant to be analysed. With the use of a freezing point depression osmometer (Osmomat 030, Gonotec, GmbBH, Berlin, Germany), saliva osmolality was determined to allow for concentration of salivary immunological parameters to be expressed relative to saliva osmolality. Aliquots of saliva were screened for blood contamination by the determination of salivary transferrin concentration using an ELISA kit (Salivary blood contamination enzyme immunoassay kit, Salimetrics, State College, Pennsylvania, USA). Samples were considered to be contaminated with blood if salivary transferrin concentration was greater than 1 mg dL^−1^, with all other samples for that subject being excluded (except SIgA due to assay being specific to secretory IgA and hence not affected by blood contamination) from the study. The concentration of SIgA and AMPs (lysozyme and lactoferrin) were determined in saliva samples using a well-established in-house sandwich ELISA technique (specific to the secretory component of human IgA) and commercially available ELISA kits (Assaypro LLC, St-Louis, MO) respectively, in accordance with Jones et al. [10].

### Sample size

A sample size of *n* = 12 was required to be able to detect at least a medium trial x time interaction effect size for fMLP-stimulated neutrophil oxidative burst, assuming a repeated measures (within subject) correlation of 0.83 [9] with 5% level of significance and 80% power. We aimed to recruit *n* = 16 for this trial to account for potential dropouts.

### Statistical analyses

Data shown in the text, tables and figures are presented as mean ± standard deviation. Statistical analysis of all data was performed via the statistical computer software package SPSS (v29.00; IBM. Armonk, NY, USA). The primary endpoint of this study was blood neutrophil stimulated oxidative burst. All data were checked for normal distribution by observations of skewness and kurtosis Z scores and tested using the Shapiro-Wilk test. Data not normally distributed were normalised with log or square root transformation before further analysis. Initially, either a two-factor repeated measures ANOVA was carried out on all immunological measures, plasma volume, plasma glucose and plasma lactate to determine if the effect of time was different between bovine colostrum and placebo (trial/group). Any significant main effects identified in the ANOVA, were further analysed by post hoc 2-tailed paired t-tests with Holm-Bonferoni correction and one way ANOVA on each trial when there was evidence of trial × time interaction. The mean values of bovine colostrum and placebo trials for nutrient intake (prior to trial) and HR, RPE and oxygen uptake ($\dot{\mathrm{V}}{\mathrm{O}}_{2}$) (during trial) were calculated and compared using 2-tailed paired t-tests. Statistical significance was accepted at *p* < 0.05.

## Results

### Characteristics of participants and main experimental trials

Sixteen recreationally active males (age: 25 ± 6 years; height: 178 ± 6 cm; body mass: 75.7 ± 7.5 kg; $\dot{\mathrm{V}}{\mathrm{O}}_{2}$_max_: 54.4 ± 9.3 ml kg^−1^ min^−1^) took part in this investigation. Details on nutrient intake in the seven days prior to main experimental trials, and oxygen uptake, HR, RPE, plasma volume, plasma glucose and plasma lactate during the trials are reported in the Supplementary material. There were no significant differences between the trials in any measure.

### Immune cell counts

A main effect of time (*p* < 0.001) was observed with all leukocyte counts (Table 1) but there was no significant trial or interaction effects (all *p* > 0.05, Table 1). There was a significant increase in total leukocytes, neutrophils, monocytes, neutrophil: lymphocyte ratio and large immature cells from Baseline to Post-exercise and 1 h Post-exercise (*p* ≤ 0.001). Total lymphocyte count at Pre-exercise was significantly lower than Baseline (*p* < 0.01) and Post-exercise (*p* < 0.001). There was a significant increase in neutrophil: lymphocyte ratio (*p* < 0.001) and decrease in total lymphocytes and monocytes (*p* < 0.01) from Post-exercise to 1 h Post-exercise (*p* < 0.01).

**Table 1:** Immune cell counts following acute bovine colostrum or placebo consumption

Cell count, 10^9^ L^−1^	Baseline	Pre-exercise	Post-exercise	1 h Post-exercise	*p* values trial time interaction
Total leukocytes			†	†	0.255
Colostrum	5.38 ± 1.46	5.51 ± 1.44	12.98 ± 5.01	12.15 ± 4.36	< 0.001*
Placebo	5.45 ± 1.33	5.17 ± 1.00	11.80 ± 3.43	11.43 ± 2.86	0.349
Neutrophils			†	†	0.566
Colostrum	2.77 ± 1.15	3.23 ± 1.28	9.38 ± 4.19	9.54 ± 3.96	< 0.001*
Placebo	2.80 ± 0.79	2.98 ± 0.83	8.56 ± 2.96	8.86 ± 2.49	0.495
Monocytes			†	† ||	0.820
Colostrum	0.50 ± 0.15	0.47 ± 0.13	0.90 ± 0.32	0.75 ± 0.23	< 0.001*
Placebo	0.53 ± 0.16	0.46 ± 0.12	0.82 ± 0.28	0.72 ± 0.19	0.211
Total lymphocytes		‡		||	0.073
Colostrum	1.86 ± 0.45	1.62 ± 0.39	2.41 ± 0.74	1.77 ± 0.27	< 0.001*
Placebo	1.89 ± 0.67	1.53 ± 0.33	2.16 ± 0.62	1.63 ± 0.39	0.176
Neutrophil: lymphocyte			†	†§	0.454
Colostrum	1.55 ± 0.67	2.13 ± 1.06	3.95 ± 1.36	5.49 ± 2.13	< 0.001*
Placebo	1.59 ± 0.59	2.03 ± 0.78	4.27 ± 2.01	5.62 ± 1.68	0.962
Large immature cells			†	†	0.870
Colostrum	0.04 ± 0.02	0.04 ± 0.02	0.20 ± 0.14	0.18 ± 0.13	< 0.001*
Placebo	0.05 ± 0.02	0.04 ± 0.02	0.17 ± 0.10	0.15 ± 0.06	0.402

*Significant main effect of time (*p* < 0.001); †Significant increase from Baseline (*p* ≤ 0.001); ‡Significant decrease from Baseline; §Significant increase from Post-exercise; ||Significant decrease from Post-exercise (*p* < 0.01)

### Neutrophil responses

**Fig. 1:**
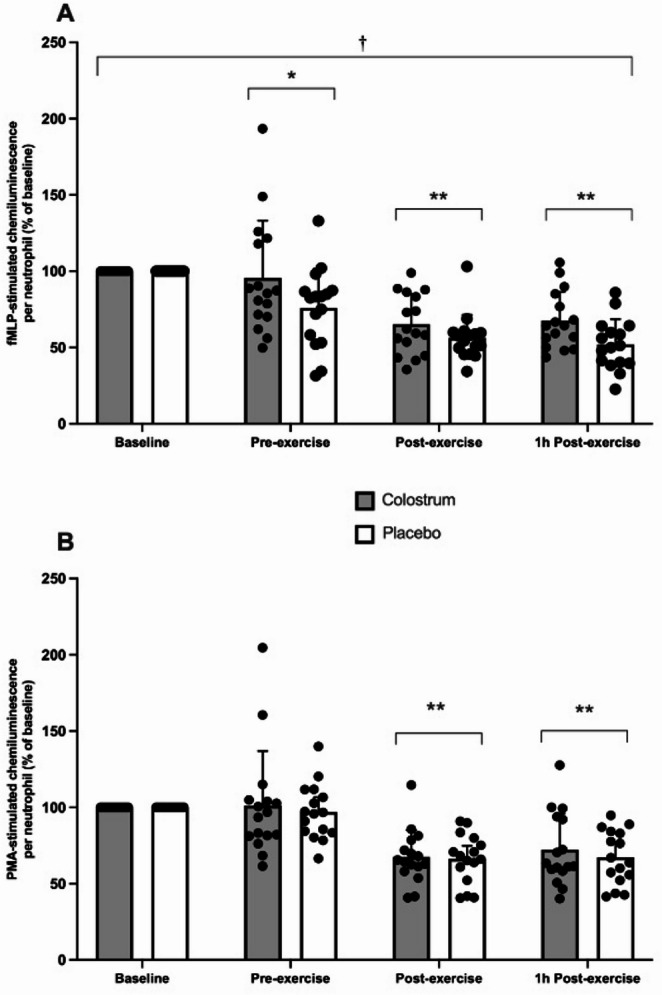
fMLP (**A**) and PMA (**B**) stimulated chemiluminescence per neutrophil following acute bovine colostrum or placebo consumption. Significant change from Baseline (*p < 0.05, **p < 0.001). †Significant main effect of trial for fMLP-stimulated chemiluminescence per neutrophil (*p* < 0.05)

For fMLP-stimulated chemiluminescence per neutrophil, there was a significant main effect of trial (*p* = 0.024), a significant main effect of time (*p* < 0.001), but no trial × time interaction (*p* = 0.158) (Fig. 1A). Post hoc analysis for the time effect showed a significant decrease in fMLP-stimulated chemiluminescence per neutrophil compared to Baseline at Pre-exercise (*p* = 0.020), Post-exercise (*p* < 0.001) and 1 h Post-exercise (*p* < 0.001).

For PMA-stimulated chemiluminescence per neutrophil, there was a significant main effect of time (*p* < 0.001) but no main effect of trial (*p* = 0.449) or trial × time interaction (*p* = 0.741) (Fig. 1B). Post hoc analysis for the time effect showed a significant decrease in PMA-stimulated chemiluminescence per neutrophil compared to Baseline at Post-exercise (*p* = 0.001) and 1 h post-exercise (*p* < 0.001).

For stimulated elastase release per neutrophil (neutrophil degranulation), there was a significant trial × time interaction, *p* = 0.01), but there was no significant main effect of trial (*p* = 0.257) or time (*p* = 0.135) (Table 2). A one-way ANOVA on each trial showed a time effect in the placebo trial (*p* = 0.009) but not in the bovine colostrum trial (*p* = 0.547). Further post hoc analysis of the placebo trial showed there was a significant decrease in stimulated elastase release from Pre-exercise to 1 h Post-exercise (*p* = 0.029).

**Table 2:** Neutrophil degranulation responses following acute bovine colostrum or placebo consumption

Measure	Pre-exercise	Post-exercise	1 h Post-Exercise	*p* values trial time interaction
Stimulated elastase release per neutrophil (fg cell^−1^)				0.257
Colostrum	401 ± 168	366 ± 143	396 ± 191	0.135
Placebo	467 ± 179	418 ± 105	356 ± 119†	0.010*

*Significant main effect of time (*p* < 0.001). †Significant main trial × time interaction (*p* < 0.05)

**Fig. 2:**
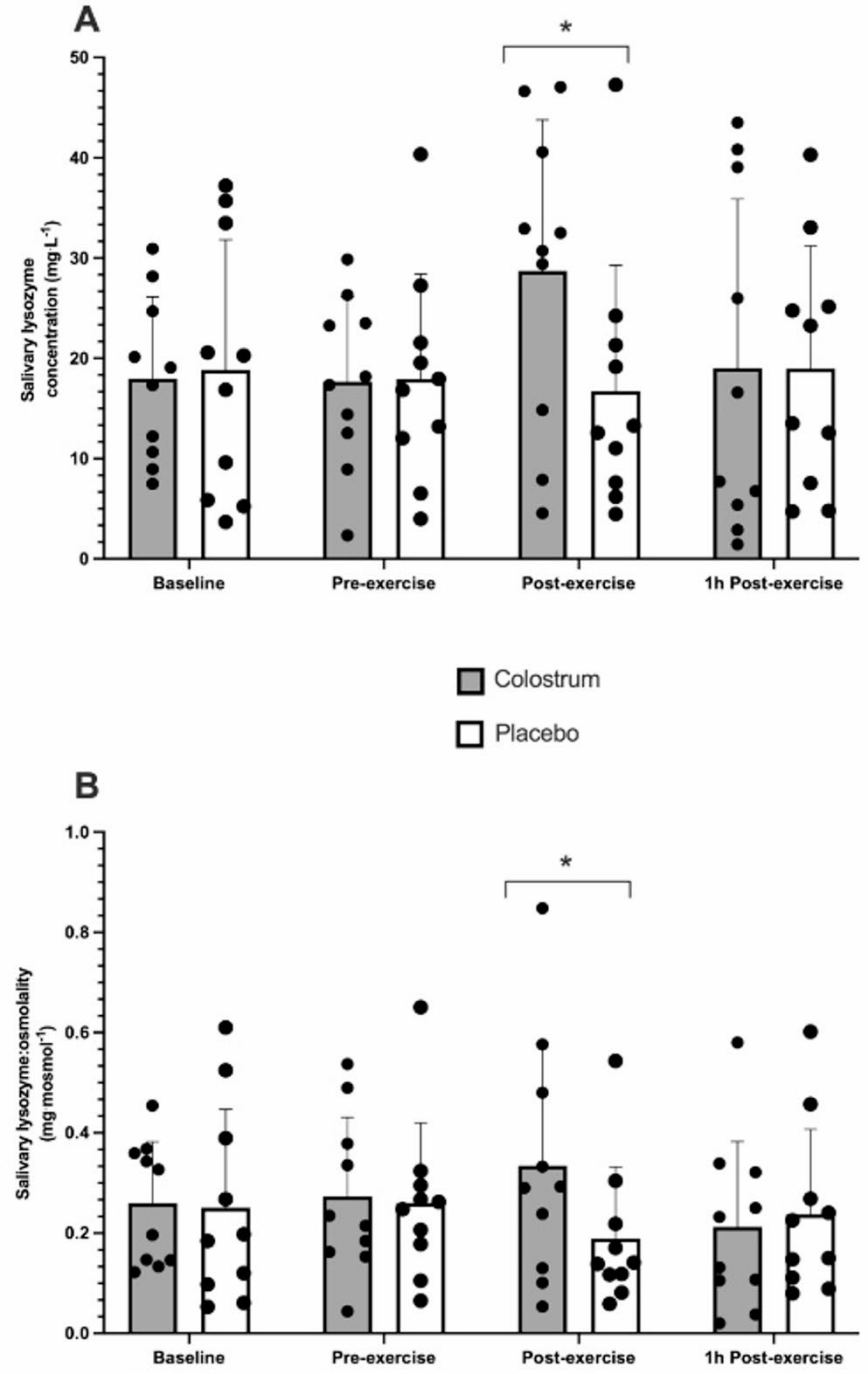
The response of sLys concentration (**A**) and sLys: osmolality (**B**) following acute bovine colostrum or placebo supplementation. *Significant difference between trials (*p* < 0.05)

### Mechanistic sub-study

**Table 3:** Chemiluminescence (oxidative burst) responses following pre-incubation of whole blood from placebo main trial with plasma collected following bovine colostrum or placebo consumption

fMLP- stimulated chemiluminescence per neutrophil (RLU s^−1^ cell^−1^)	Baseline	Post-exercise	*p* values trial time interaction
			0.026*
Colostrum	32.8 ± 12.1	24.0 ± 10.7	0.087
Placebo	25.3 ± 13.7	21.0 ± 9.1	0.293

*Significant main effect of trial (*p* < 0.05)

Two-way ANOVA analysis revealed a significant main effect of treatment for fMLP-stimulated oxidative burst (*p* = 0.026) but no effect of time (*p* = 0.087) or treatment × time interaction (*p* = 0.293). The observed effects indicated an overall higher response to fMLP across both timepoints (Baseline and Post-exercise) when whole blood was pre-incubated with plasma obtained 1 h following bovine colostrum consumption (Table 3). It is worthy to note that the main focus here was to observe the main effect of bovine colostrum as the temporal pattern (i.e. interaction effect) may be confounded by enhancements with bovine colostrum at both timepoints and a lack of statistical power compared to the main experimental trial to identify an effect of time (i.e. exercise).

### Saliva SIgA and antimicrobial peptide responses

**Table 4:** Changes in salivary SIgA and antimicrobial peptides with bovine colostrum or placebo supplementation

Immune measure	Baseline	Pre-exercise	Post-exercise	1 h Post-exercise	*p* value trial time interaction
SIgA concentration (mg L^−1^)		†			0.604
Colostrum	364 ± 177	317 ± 201	359 ± 164	350 ± 191	0.002*
Placebo	385 ± 183	300 ± 151	352 ± 174	374 ± 163	0.518
SIgA secretion rate (µg min^−1^)					0.384
Colostrum	160 ± 95	196 ± 116	179 ± 119	191 ± 104	0.315
Placebo	161 ± 106	159 ± 94	179 ± 132	194 ± 118	0.220
SIgA: osmolality (mg mosmol^−1^)			†		0.205
Colostrum	5.3 ± 1.8	4.8 ± 2.6	4.1 ± 1.2	4.5 ± 1.8	< 0.001**
Placebo	5.4 ± 1.8	4.6 ± 1.7	4.2 ± 1.3	5.2 ± 1.6	0.150
sLac concentration (mg L^−1^)			‡		0.602
Colostrum	3.9 ± 0.8	4.0 ± 0.9	4.9 ± 1.0	4.3 ± 1.1	0.001*
Placebo	3.7 ± 1.0	4.3 ± 1.0	4.9 ± 0.9	4.5 ± 1.1	0.327
sLac secretion rate (µg min^−1^)					0.549
Colostrum	2.3 ± 1.5	3.2 ± 2.0	2.9 ± 1.7	3.1 ± 1.8	0.044*
Placebo	2.0 ± 1.6	3.1 ± 1.9	2.8 ± 1.5	2.7 ± 1.3	0.813
sLac: osmolality (mg mosmol^−1^)					0.855
Colostrum	0.06 ± 0.01	0.06 ± 0.01	0.05 ± 0.02	0.06 ± 0.02	0.022*
Placebo	0.05 ± 0.02	0.06 ± 0.01	0.06 ± 0.01	0.06 ± 0.02	0.067
sLys secretion rate (µg min^−1^)					0.528
Colostrum	10.0 ± 7.3	12.9 ± 7.1	17.2 ± 13.4	12.6 ± 12.0	0.254
Placebo	9.8 ± 11.5	11.9 ± 8.2	8.8 ± 6.7	11.4 ± 8.2	0.061

Significant main effect of time (* *p* < 0.05, ** *p* < 0.001). †Significant decrease from Baseline (*p* ≤ 0.001); ‡Significant increase from Baseline and Pre-Exercise (*p* < 0.05).

For salivary SIgA concentration, there was a significant main effect of time but there was no main effect of trial (*p* = 0.604) or interaction (*p* = 0.518) (Table 4). Post hoc analysis of the main effect of time (*p* = 0.002) showed that salivary SIgA concentration was lower at Pre-exercise compared to Baseline (*p* = 0.006) and Post-exercise (*p* = 0.039). For salivary SIgA secretion rate, two-way ANOVA revealed no significant main effects of time (*p* = 0.315), trial (*p* = 0.384) or interaction (*p* = 0.220) (Table 4). For saliva SIgA: osmolality, there was a significant main effect of time (*p* < 0.001) but no main effect of trial (*p* = 0.205) or interaction (*p* = 0.150). Post hoc analysis of the main effect of time showed a significant decrease in SIgA: osmolality from Baseline to Post-exercise (*p* < 0.001).

Blood contamination was detected in the saliva samples from six participants, leaving *n* = 10 for analysis of salivary lysozyme (sLys) and lactoferrin (sLac). For sLys concentration, two-way ANOVA showed a significant interaction (trial × time) (*p* = 0.018) but no main effect of time (*p* = 0.333) nor trial (*p* = 0.059) (Fig. 2A). Post hoc paired t-tests showed there was a higher sLys concentration at Post-exercise in the bovine colostrum trial compared to placebo (*p* = 0.007). This was supported by a one-way ANOVA on each trial showing a main effect of time (*p* = 0.026) with significantly greater sLys concentration at Post-exercise compared to Pre-exercise in bovine colostrum trial (*p* = 0.031) while there was no significant change across time for sLys concentration in the placebo trial (*p* = 0.766). For sLys secretion rate, rhere was no statistically significant (*p* = 0.061) interaction (trial × time) or main effect of trial (*p* = 0.254) or time (*p* = 0.528) (Table 4). For sLys: osmolality, two-way ANOVA showed a significant interaction (trial × time) (*p* = 0.042) but no significant main effects of trial (0.226) or time (0.356) (Fig. 2B). Post-hoc paired t-tests showed there was a higher sLys: osmolality at Post-exercise in the bovine colostrum trial compared to placebo trial (*p* = 0.013). One-way ANOVA on each trial revealed no significant main effects of time in the bovine colostrum (*p* = 0.093) or placebo trials (*p* = 0.312).

For sLac concentration, there was a significant main effect of time (*p* = 0.001) but no significant main effect of trial (*p* = 0.602) or interaction (*p* = 0.327) (Table 4). Post-hoc analysis of the main effect of time showed that sLac concentration was increased at Post-exercise compared to Baseline (*p* = 0.033) and Pre-exercise (*p* = 0.039). for For sLac secretion rate, there was a significant main effect of time (*p* = 0.044) but no effect of trial (*p* = 0.549) or interaction (*p* = 0.813). Post-hoc analysis of the main effect of time could not identify any statistically significant differences between time points (Table 4). For sLac: osmolality, there was a main effect of time (*p* = 0.022) but no main effect of trial (*p* = 0.855) or interaction (*p* = 0.067). Post hoc analysis of the main effect of time could not identify statistically significant differences between timepoints (Table 4).

## Discussion

The main finding of this study was that acute consumption of bovine colostrum resulted in a greater fMLP-stimulated neutrophil oxidative burst compared to placebo. Acute bovine colostrum consumption prior to a prolonged exercise bout also results in greater bacterial-stimulated neutrophil degranulation and salivary lysozyme post-exercise. This supports previously mentioned benefits of daily bovine colostrum supplementation on the human innate immune system following prolonged exercise [8, 9], but this is the first study to demonstrate such effects with acute consumption.

Similar to previous studies of daily bovine colostrum supplementation (10 days to 4 weeks) the current study observed no significant effect of acute bovine colostrum on the pronounced increases in total leukocytes, neutrophils, lymphocytes (followed by lymphocytopenia), neutrophil: lymphocyte ratio, and monocytes induced by prolonged exercise [8, 9, 23]. The current study lends support to previous proposals that immune-enhancing effects of bovine colostrum are due to ‘priming’ and/or immune-enhancing components that directly affect leukocyte function [8, 15]. The mechanistic sub-study showing enhancement of oxidative burst when neutrophils are pre-incubated with plasma collected following acute bovine colostrum consumption, but not placebo, supports the view that such components have become biologically available to affect neutrophil function.

Evidence from numerous in vitro culture studies suggest that brief exposure to bovine colostrum primes the responses of neutrophils to subsequent stimulation [12–15]. Sugisawa et al. [15] proposed that priming effects are due to low molecular weight components such as proteose peptones following findings that a bovine colostrum fraction containing substances less than 10 kDa enhanced responses to *Staphylococcus aureus* whereas fractions containing constituent cytokines (IL-1, IL-6, TNFα) of bovine colostrum (also capable of priming neutrophils) did not have any effects. Bovine colostrum is also considered to be a precursor of numerous potential biologically active peptides [24]. For example, although IgG in the bovine colostrum supplement is unlikely to pass into circulation, the digestion of its Fc heavy chain may release other metabolites which have been shown to modulate neutrophil functions. Mechanism(s) triggered by metabolites of bovine colostrum would challenge hypotheses proposed by others that the component is present within whole bovine colostrum. The direct or indirect effects of bovine colostrum on cytokines (also capable of modifying neutrophil responses) in vivo cannot be excluded. Of note to the current study is previous in vitro culture studies have demonstrated that the priming effects of bovine colostrum were absent when neutrophils were stimulated with PMA [15] or that PMA-stimulated oxidative burst responses were mild and substantially lower compared to responses to bacterial (*Staphylococcus aureus*) stimulation [13]. Enhancement of PMA-stimulated oxidative burst with bovine colostrum consumption in a resting state in the pilot investigations for the current study was not replicated in the exercise trials. Further studies are required to elucidate the specific mechanisms of bovine colostrum but we suggest that components of the supplement and/or its metabolites possess the ability to prime neutrophil responses by mediating various pathways of signal transduction but the functional significance of these may be influenced by exercise.

Like Davison and Diment [8] but not Jones et al. [9], bovine colostrum compared with placebo enhanced sLys concentration immediately-post exercise, before the parameter returned to pre-exercise levels in both trials after 1 h of recovery. Such findings may add further supporting evidence to the aforementioned proposals of bovine colostrum and the effector responses of neutrophils. In addition to circulating neutrophilia, it has been suggested that there is an increase in the number of neutrophils in mucosal secretions following prolonged exercise [25]. As neutrophils are considered an important source of sLys [18], it is plausible that the increased concentrations of lysozyme post-exercise are due to the migration of ‘primed’ neutrophils into the oral cavity. sLys along with sLac are the most abundant AMPs within saliva [26]. In contrast to sLys, there was increased sLac concentration post-exercise in both the bovine colostrum and placebo trials. These exercise-induced changes, however, agree with other investigations of sLac or other AMPs that have similar sources (e.g. LL37) (submucosal glands and epithelial cells) [19, 27]. The functional significance of these increases is unclear at present, but it may reflect an inflammatory response to exercise-induced damage to mucosal surfaces. Given the suggested synergistic effects of these AMPs on host defence within the oral cavity, further research is required to elucidate any influence by bovine colostrum. There was also no apparent effect of bovine colostrum on salivary SIgA in this study. Within this study design, benefits to salivary SIgA may have been via a modulation of responses to exercise or an elevation in pre-exercise levels. Our study adds to the growing number of studies of bovine colostrum that have not shown any effect on SIgA. Although increases in resting salivary SIgA with bovine colostrum have been observed in some studies [28, 29], the body of available evidence suggests that changes in SIgA do not represent the key immune-modulatory mechanism of colostrum supplementation.

A possible limitation to the present study may be that there was a lack of or minimal exercise-induced immune dysfunction in some parameters (e.g. salivary SIgA) providing little scope for benefit via a nutritional countermeasure (i.e. bovine colostrum). Despite significant falls in blood neutrophil oxidative burst following prolonged exercise, the lack of simultaneous decrease in bacterial-stimulated degranulation immediately post-exercise is somewhat surprising and dissimilar to many previous findings (e.g. [30, 31]). It is likely that the cumulative intensity and duration of the current exercise protocol was not strenuous enough in the study population to trigger decreases in neutrophil degranulation, where decreases in neutrophil capacity are reported to be associated with increasing neutrophilia (i.e. greater physiological stress) [32]. The lack of measurement of hormonal (adrenaline, cortisol, and inflammatory mediators, e.g. IL-6) limits our ability to specifically compare the physiological stress of the current study to previous studies. Furthermore, while such markers may provide additional mechanistic insight, previous work suggests that systemic inflammatory cytokines are unlikely to represent a primary pathway underpinning the effects of bovine colostrum supplementation in this context [5, 6, 8, 9].

Whilst this study alone cannot fully define the optimal dosage of colostrum supplementation to enhance immunity in populations undergoing strenuous exercise, it does provide important new evidence to help inform dosage schemes. Prior studies have utilised daily bovine colostrum supplementation (e.g. 20 g) for 4 weeks or longer and have shown benefits on in vivo immune responsiveness to a novel antigen, ex vivo measures of innate immune cell function (neutrophil oxidative burst, neutrophil stimulated-degranulation) and secretion of salivary antimicrobial peptides (lysozyme) [5, 6, 8–10). All of these studies involved supplementation prior to the day of exercise, and the bout of prolonged exercise completed in a fasted state. The benefits on innate immune cell function and salivary antimicrobial peptides from 4 weeks of supplementation (20 g daily, [6, 8, 9]) were larger than those observed with acute supplementation (30 g 1 h prior, 5 g immediately prior and 5 g during exercise) in the current study, but the latest findings demonstrate the benefits with acute consumption. It is unclear whether these acute effects would be observed in addition to any prior daily supplementation (e.g. 4 weeks). Based on these findings, it appears that daily supplementation for several weeks may be optimal as a nutritional countermeasure to exercise-induced immune dysfunction, but there are no full dose-response studies with a full range of different doses compared in the same study. Likewise for acute intake, whilst the findings show that benefit can be gained from acute intake on the day of exercise, further trials are required to define the optimal acute dosage. Although no acute dose–response trials exist to determine this, the available evidence suggests that acute intakes are also likely to require at least ~ 20 g to elicit measurable benefits.

To conclude, this is the first to study to demonstrate that acute bovine colostrum supplementation can enhance components of the innate immune system following prolonged exercise. It is proposed that this is primarily due to priming agents that become bioavailable systemically upon bovine colostrum consumption. Further randomised trials are required to confirm this and will require metabolomic and proteomic approaches to fully explore the mechanisms of effect.

## Supplementary Information

Below is the link to the electronic supplementary material.

Supplementary Material 1

## Data Availability

Data are available from the following public data repository: https://data.kent.ac.uk/584/
