# Supplementary material for: Effects of acute bovine colostrum supplementation on immune responses to prolonged cycling: a randomised crossover trial
Source: Eur J Nutr. 2026 Jun 6;65(4):153. doi: 10.1007/s00394-026-04016-5 (PMC13242472; doi:10.1007/s00394-026-04016-5)
Supplement: Supplementary file 1 — Supplementary Material 1 [file 394_2026_4016_MOESM1_ESM.docx]

**Pilot study**

Pilot work was carried out to determine a duration and dosage of bovine colostrum that could potentially act as an acute intervention in human volunteers under conditions of exercise-induced immune dysfunction. Following an overnight fast, 3 males reported to the laboratory on two occasions separated by seven days. On each occasion, participants either consumed 30 g of bovine colostrum or 30 g of placebo (isoenergetic mixture of milk protein concentrate and skimmed milk powder) mixed in 300 mL water in a randomised double-blind manner. Solutions were also flavoured with vanilla flavouring (MP flavouring, My Protein, Northwich, UK) and were indistinguishable in flavour or appearance. Resting blood samples were taken from an antecubital vein immediately prior (Baseline) to consumption of bovine colostrum or placebo as well as 1 h and 3 h post-consumption. Participants remained fasted until after the 3 h blood sample was taken. At each timepoint, PMA-stimulated oxidative burst and neutrophil count was assessed in whole blood collected from the K_3_EDTA vacutainer. This preliminary work suggested that bovine colostrum may have a direct, acute effect on the functional capacity of blood neutrophils (Table S1). Compared to placebo, a greater increase in stimulated oxidative burst with bovine colostrum was evident at 1 h post consumption which was maintained for a further 2 h later. A larger scale study was then completed to determine whether these benefits on neutrophil function are observed following acute bovine colostrum supplementation under conditions of exercise-induced perturbations in innate immunity.

**Table S1**. Pilot study to assess acute effects of colostrum on blood neutrophil function at rest.

| **Supplement** | **PMA-stimulated chemiluminescence per neutrophil (% of baseline)** | | |
| --- | --- | --- | --- |
|  | **Baseline** | **1 h** | **3 h** |
| Colostrum | 100 ± 0 | 133 ± 31 | 134 ± 29 |
| Placebo | 100 ± 0 | 92 ± 32 | 110 ± 38 |

**Main experimental trials**

**Results**

**Nutrient intake**

In the seven days prior to the two main experimental trials, there was no statistically significant difference in total energy (p = 0.443) and macronutrient intake of the participants (carbohydrate: p = 0.318; fat: 0.554; protein: p = 0.891). The mean daily macronutrient intake (as a percentage of total energy intake) prior the bovine colostrum trial was carbohydrate 52.1 ± 5.9%, fat 32.3 ± 7.0% and protein 15.6 ± 3.9%. Prior to the placebo trial the mean daily macronutrient intake was carbohydrate 50.9 ± 7.2%, fat 32.9 ± 6.3 % and protein 15.8 ± 3.3%.

**Physiological variables and perceived exertion**

There was no significant difference in oxygen uptake () (p = 0.218; p = 0.138) between the bovine colostrum (2336 ± 279 mL·min^-1^; 57.0 ± 4.2% max) and placebo trial (2297.8 ± 274.6 mL·min^-1^; 55.8 ± 4.5% max). The average HR (134 ± 15 bpm and 132 ± 14 bpm during bovine colostrum and placebo trials respectively) was not significantly different (p = 0.209). There was no significant difference in average RPE (p = 0.174) between the bovine colostrum (12.9 ± 1.3) and placebo (12.6 ± 0.8) trials. A similar pattern of plasma volume changes was observed from Baseline between trials: bovine colostrum; Pre-exercise (0.5 ± 4.3%); Post-exercise (-3.7 ± 5.6%); 1h Post-Exercise (-0.1 ± 4.5%) and placebo; Pre-Exercise (-1.7± 2.1%); Post-exercise (-5.4 ± 3.1%), 1h Post-Exercise (-2.0 ± 5.6%). As there was no significant difference between trials (trial × time interaction; p = 0.915), it was deemed unnecessary to correct any haematological parameters for plasma volume changes.

Although there was a significant change across time (p < 0.001), there was no significant trial (p = 0.073) or interaction effect (p = 0.694) on plasma glucose (Table S2). Plasma glucose was significantly lower at Pre-exercise (p < 0.001), Post-exercise (p = 0.003) and 1h Post-exercise (p < 0.001) compared to Baseline. There was also a significant change across time for plasma lactate (p < 0.001) but no effect of trial (p = 0.970) or interaction (p = 0.381) (Table S2). Plasma lactate significantly increased from Baseline (p = 0.001) and Pre-exercise (p < 0.001) to Post-exercise before decreasing towards resting levels. at 1h Post-exercise (p = 1.000).

**Table S2**. Plasma glucose and lactate responses following acute colostrum or placebo supplementation.

|  | **Baseline** | **Pre exercise** | **Post-exercise** | **1h post exercise** | **p values**  **trial; time; interaction** |
| --- | --- | --- | --- | --- | --- |
| **Plasma glucose, mmol⋅L^-1^** | |  |  |  | 0.073; < 0.001*; 0.694 |
| Colostrum | 5.10 ± 0.39 | 4.39 ± 0.57 | 4.38 ± 0.42 | 4.25 ± 0.39 |  |
| Placebo | 5.19 ± 0.51 | 4.43 ± 0.55 | 4.56 ± 0.53 | 4.35 ± 0.34 |  |
| **Plasma lactate, mmol⋅L^-1^** | |  |  |  | 0.970; < 0.001*; 0.381 |
| Colostrum | 1.50 ± 0.48 | 1.37 ± 0.29 | 2.04 ± 0.71 | 1.69 ± 0.66 |  |
| Placebo | 1.49 ± 0.38 | 1.42 ± 0.26 | 1.97 ± 0.69 | 1.60 ± 0.43 |  |
